# At the edge of vulnerability—lived experience of parents of children with cerebral palsy going through surgery

**DOI:** 10.3402/qhw.v8i0.20007

**Published:** 2013-02-06

**Authors:** Anne Solveig Iversen, Marit Graue, Målfrid Råheim

**Affiliations:** 1 Department of Nursing, Faculty of Health and Social Sciences, Bergen University College, Bergen, Norway; 2 Centre of Evidence-Based Practice, Faculty of Health and Social Sciences, Bergen University College, Bergen, Norway; 3 Department of Pediatrics, Haukeland University Hospital, Bergen, Norway; 4 Department of Global Public Health and Primary Care, University of Bergen, Bergen, Norway

**Keywords:** Parenting, children, cerebral palsy, lived experience, surgery, hospitalization, hermeneutic phenomenology

## Abstract

This study explored the experiences of parents of children with cerebral palsy undergoing surgery as they describe them from a lived experience perspective. When children undergo surgical procedures, they have to stay at hospital for a long time, which represents a great challenge for the children as well as their parents. We collected data by using open-ended interviews with 12 parents of 9 children and analyzed these data in accordance with Max van Manen's methodological themes. Based on the parents’ stories, the essential theme is: At the edge of vulnerability—being parents at hospital to a child with CP undergoing surgery, which consisted of three subthemes: establishing trust, awareness of a child who cannot speak, and sensing bodily reactions. Parents experienced demanding challenges as they entered the hospital, in a situation that meant both familiarity and unfamiliarity. Judgments about how to care for the child relied on what they normally did. Sitting bedside for hours and days, thoughts about the legitimacy of letting their child go through the suffering surgery were tormenting the parents. They felt vulnerable and very much dependent on health care workers’ competence and at the same time doubting them in seeing and taking care of their child's specific needs. It was experienced as an ambivalent situation, and even more so for the parents of a child without speech. The findings indicate that establishing trust implies being met at an existential level and a deeply felt need for health care workers that are really engaged in taking care of their child and their parents.

Cerebral palsy (CP) is the most common reason for motor impairment in children (Åkerstedt Risto, Ødman, & Øberg, 2010). Conditions which result from spasticity such as scoliosis, hip dysplasia, ankle deformities, and joint contractures are frequented treated by orthopedic procedures (Knaus & Terjesen, 2009; McKearnan, Kieckhefer, Engel, Jensen, Labyak, 2004; Murphy et al. 2006). Children undergoing these surgical procedures have to stay at hospital for a long time, which represents a great challenge to the children as well as their parents.

Children with CP are often hospitalized many times during their first years of life, and most have been in contact with the health care system in various contexts (Bø & Olsen, 2008; Wigert, Berg, & Hellstrøm, 2007). Having a disabled child in hospital is a major source of stress, and the child's illness and hospitalization invariably stir up intense emotions (Hopia & Tomlinson, 2005; Iversen, Graue, & Clare, 2009; Secco, Askin, Ecco, & Yu, 2006). When critically ill children are hospitalized, it is shown that the parents experience emotional disturbances such as anger, guilt, sadness, and loneliness (Hopia, Paavilainen, & Åstedt-Kurki, 2005). For children too, hospitalization creates feelings of anger and fear, affecting their condition and behavior, which then affects how the parents feel about the whole situation (Holaday, Hallstrøm, Runesson, & Elander, 2002). A study of the experience of the parents of critically ill newborns or young children found that being a parent in this situation resembles being in another world, alien from earlier experiences (Hall, 2005). They expressed a strong need to stay at the hospital, monitoring the child closely, and felt safer when they could care for the child themselves. The study of Hopia et al. (2005) emphasized that the parents of children with chronic illness were not sure about their role at the hospital. The parents often felt insecure and helpless and needed positive feedback on their parenthood. They expected nurses to know about the child's home life and personality. In the hospital, the child often had to cope with pain and difficult procedures, and parents had to rely on help from nursing staff when the child showed behavioral changes and emotional outbursts. Parents wanted to share their emotional burden and expected practical help surrounding the care and treatment of their child. A care relationship based on confidence is shown to be important, because parents may also return to earlier experiences of the child's illness and need help in working through reactivated experiences (Kristensson-Hallström, 2000; Peden-McAlpine, Tomlinson, Forneris, Genck, & Meiers, 2005).

The parents of a child with severe disabilities have an extremely tough life (Brinchmann, Førde, & Nortvedt, 2002), and the relationship with the child may be ambivalent. Children with CP are very dependent on their parents, and the parents have continuous tasks and responsibilities, getting little rest and sleep. In addition, CP is often accompanied by associated impairments, which affect communication. Children who speak poorly or not at all have communication problems, both with parents, and others (Bø & Olsen, 2008; McCullough, Parkes, Kerr, & McDowell, 2011). Parents have reported that they feel vulnerable because their children are helpless and in need of constant attention (Brinchmann et al., 2002; Iversen et al., 2009; Secco et al., 2006). Being a parent of a child with CP indicates entering a difficult emotional area and comprehending that one's child differs from other children. Hence, the parents of these children are probably vulnerable when entering the hospital. Studies about parents’ perspectives have mostly focused on parents of critically ill children. However, research on the experiences of the parents of children with disabilities undergoing hospitalization is sparse. To improve how pediatric care is delivered, health care providers need to know what both children and parents need, expect, and experience (Kristensson-Hallström, 2000; Huang, Kellett, & St. John, 2010; Wigert et al., 2007). This study explored the lived experiences of parents of children with CP undergoing surgery, as they describe them.

## Methods

The study is grounded in hermeneutic phenomenology, as outlined by van Manen (1997), a method that is both descriptive and interpretive. Making visible the essential dimensions of the lived experience of parents as they care for their child with CP at hospital means going beyond merely structuring what is said to a deeper level of interpretation (Jones & Borbasi, 2003; Kvale, 2005; van Manen, 1997).

The hermeneutical or interpretive endeavor of the method refers to pre-understandings at play, which are grounded in the history and tradition to which one belongs (Gadamer, 1993). Gadamer (1993, in Nystrøm & Dahlberg, 2001, p. 340) declares that pre-understanding is an intentional structuring of feelings and thoughts that are activated when people regard something as something. Following Heidegger, this understanding of each other is not neutral or distanced, but engaged and in need of sensitivity (1962, in Vetlesen & Stanicke, 1999, p. 152). According to van Manen, a phenomenological attitude means to make explicit understandings and beliefs, and purposefully seek an attitude of wonder and thoughtful dwelling in the process of trying to understand another person and the phenomenon under study (1997, p. 12). Thus, pre-understanding is both a prerequisite for and a possible obstacle to new understanding. The first author conducted the interviews. She is an experienced nurse in postoperative care and a teacher in pediatric nursing.

### Choice of method for data production

An appropriate method of obtaining rich descriptive data is in-depth interviews, in which the researcher actively listens to people as they explore the meaning of their experiences (Kvale 2005). We found in-depth interviews well suited to search for the deeper meanings of the parents’ experiences when caring for their child at hospital.

### Recruiting the participants

The surgeon in the department of the hospital where the child was hospitalized arranged contact with the head nurse. The head nurse of the department made contact with the parents and gave them written information about the study at the hospital. Three families whose child had recovered from surgery were recruited from outside the hospital.

### Participants

The empirical material consisted of interviews with the parents of nine children with CP (8–16 years old), covering different backgrounds and experiences. All nine children lived with their parents. Three children were almost without speech, and strongly mentally retarded. Two children were difficult to understand in their way of talking, they were mentally retarded and mostly sitting in a wheelchair. The last four were partly mobile and attended an ordinary school with extraordinary resources because of learning difficulties. All parents had more children, and everyone except one mother was still married to the other parent of the child. In three of the interviews, the parents consisted of both mother and father, in three only the mother and in the last three only the father (total: 12 parents).

### Data production

The interviews took place in 2004–2005 during or after recovery from surgery. Each of the 12 parents participated in one audiotaped, open-ended interview. Six interviews were carried out in the hospital and three in the homes of the participants, in different parts of the country. The open-ended interview method afforded parents the opportunity to use their own words when talking about their experiences. A broad interview guide was developed based on topics found by reading relevant literature and based on the first author's experience in working with the parents of children with chronic illness. The interviews were produced in dialogues between the first author and the participants, forming questions along the way. Examples of questions included: “What is it like to be parents when your child is undergoing surgery?”, “Tell me what happened?”, “What did you think/feel about …?”, and “Could you tell me a bit more about …?” The interviews lasted from 1 to 1.5 h.

### Analysis

The first author transcribed the interviews verbatim, and wrote down the themes arising when reading and rereading the interview text as a whole. The analysis involved holistic reading with a thematic dimension of inquiry, and asking “What does this text speak about? as recommended by van Manen's (1997, p. 345). Preliminary identification of and reflection on themes and their meanings took place for each interview, and before analysis across the interview texts. Analysis across interview texts meant identifying and reflecting on essential themes growing from the texts. It is necessary to determine the themes around which the description will be woven (p. 106), and to differentiate between essential themes and subthemes. We were able to identify one essential theme across the interviews, consisting of three subthemes.

Included in holistic reading was analysis according to the general principle of the hermeneutical circle, in which the meaning of the text is established through an analytical process involving dynamic movements from parts to the whole and vice versa. The aim of these repeated dynamic movements was to identify and reflect on essential themes and subthemes. Discussions took place with the co-authors, who read some of the interview texts, and essential subthemes were identified and agreed on.

Listening, reading, and writing are deeply interwoven analytical processes (van Manen, 1997, p. 131). The first author asked several times about the significance of the spoken words about being parents at hospital and how to express essential meanings of their lived experience. Writing and re-writing means reflecting on lived experience and is a core dimension in the analytic process (van Manen 1997, p. 131), which was practiced in this study. According to van Manen (1997) and Merleau-Ponty (1996), constantly asking for a deeper understanding of the lived experience means being emotionally present in the situation and directed towards the phenomenon explored, in our case towards the lived experience of being parents of a hospitalized child with CP. Researchers must be sensitive to the undertone of the spoken word, and the words have to allow the phenomena themselves to speak (van Manen 1997, p. 131). In our case, the presentation of the lived experience of the parents has to express the vulnerability, exhaustion and love the parents described in the interviews. From the perspective of the researcher, this meant relating to the parents experiences at an existential level.

### Ethical implications

The Regional Committee for Medical Research Ethics approved the study. Anonymity, voluntary participation, and informed written consent were important ethical prerequisites. The parents were also informed that they could withdraw from the study with no consequences for the child's treatment and follow-up. In-depth interviews require that the researcher is sensitive and treat the participants with respect. In conversations with the parents, the first author had to be aware of the parents’ vulnerability. Researchers are not just part of a situation but have to be aware of their responsibility for the climate in the situation (Fog, 2004). Building trust is a prerequisite for sharing experiences and thoughts.

## Lived experience of being parents at hospital—findings

Lived experience of being parents at hospital was identified with the essential theme: At the edge of vulnerability—being parents at hospital of a child with CP undergoing surgery, consisting of three subthemes: establishing trust; awareness of a child who cannot speak; and sensing bodily reactions.

### At the edge of vulnerability—being parents at hospital to a child with CP undergoing surgery

Parents experienced profound challenges as they entered the hospital. At home, they were usually busy with practical tasks of caring for their child, which indicated little room to reflect upon their situation and their history as parents of a disabled child. When their child underwent surgery, they suddenly found themselves in a situation in which it was difficult to act and take care of him or her, and hence difficult to experience themselves as competent parents. These children went through quite comprehensive operations. The postoperative phase implied pain and distress, and they had to stay for a long time at hospital. Sitting bedside for hours and days, observing their child in this situation, thoughts about the legitimacy of letting their child go through the suffering surgery were tormenting the parents. Besides, the situations at hospital meant stirring up emotions from earlier hospitalizations of their child. They felt vulnerable and very much dependent on health care workers’ competence. At the same time, the parents doubted them in seeing and taking care of their child's specific needs, as well as doubting them to recognize how demanding it was to be parents to a disabled child at hospital. It was experienced as an ambivalent and precarious situation, and even more so for the parents of a child without speech. They wanted to be involved and teach the health care workers about their child's specific needs and interpretation of the child's bodily reactions, but also to be de-burdened of the responsibility for the child's well-being, as to pain medication and so on. They felt the child's bodily reactions in their own bodies, in addition to the distress they already had as worried parents in this particular situation. Establishing trust between health care workers and themselves was essential to them, which needed to be seen and met at an existential level.

### Establishing trust

Entering the hospital meant summoning pre-understandings of being ever-present caretakers, with increased sensitivity toward the child's specific needs and feeling continuously responsible. To establish trust between themselves and the health care workers in this situation meant to be seen and heard as different from parents of non-disabled children. Their children were not only physically disabled, but also more or less mentally retarded which require involvement and presence from the nurses to interpret what is going on. Some did not speak at all, others had speech, but still reduced intellectual capacity. Parents felt that nurses did not take the necessary time to get to know the child. For instance, a mother had a feeling of not being taken seriously when she wanted to know in detail what would happen with her child during hospitalization, and experienced that health personnel did not recognize how complex the whole situation was, both at the hospital and during rehabilitation. In another situation, a child got an infected wound after discharge and had to seek care at the local hospital. New nurses dressed the wound daily, but it gradually worsened. In this situation, the mother did not trust the personnel, as they did not document the worsening of the wound. She felt that they did not communicate the seriousness of the worsening wound, and related it to her child's inability to express pain and discomfort. “Health professionals have to be honest. I had to fight for my child at the local hospital. I became very tired being in that situation,” she reported.

In addition, experiences from earlier hospitalization colored their stay. To establish trust, parents had to get a feeling of nurses who wanted to take care of the child and them at a deeper level, which had not always been the case. The parents felt that they were given too much responsibility for the care of their child, which implied feelings of helplessness and vulnerability. Sitting bedside for hours and days, without rest and nobody but themselves to share their worries, they felt let down by professionals who did not ask how they could support and help. One mother said:

Our child has gone through three surgical interventions during the last three years, all with serious complications. As a family we have gone through this together. During the last intervention we experienced lots of frustration during unstable periods in our child's postoperative condition. We were actually afraid of losing her. In this situation, we experienced that the doctors tried to escape us when we wanted to talk about it.

Earlier disappointments seemed to increase distress in the current situation, underpinning their need for health professionals to ask them how they were doing, and how things usually worked out for the child. No one, except for the parents, knew the history of hospitalization or the child's present situation.

Situations of confusion were also described, for instance, when parents considered that nurses had little knowledge in general about children with CP. Professionals simply did not quite know how to take good care of their child, such as not administering adequate painkillers. In such a situation, the parents felt mistrust and sadness. One mother said:

Our son experienced too much pain, and he became angry. The child had an epidural catheter and a peripheral venous catheter for administering analgesic medicine. The nurses did not quite know the dose, and the doctor was busy in the operating room and could not prescribe medicine, as he had not seen the child. In this situation, I think my son had too much pain. After he came home, he talked a lot about the doctor who did not give him enough medicine.

Another aspect was previous experiences of not being heard at all, in contrast to feeling welcome and listened to now, which implied trust in this particular situation. One mother said:

Now we feel welcome at the hospital; all workers are kind to us. We feel respected. However, when our daughter was three years old, she was operated on. The doctors claimed that they knew what was best for our child – in every situation! Encounters were characterized by strong discipline, and the child was simply afraid of everything going on. Luckily, later we met other doctors who related to our child and us in ways that made us feel respected. Now we are good friends.

When parents observed nurses relating to their child in a humorous way, or sharing his or her interest in, for instance, football and so on, their child seemed to be able to shift focus from pain and suffering. Trust was established, between the child and the health care worker, between the parents and him/her. Parents urgently wanted health professionals to really attend to their child, and listen to the parents when they tried to share their knowledge about their child, so that the professionals could take care of their children properly.

### Awareness of a child who cannot speak

Three children could not speak at all, which virtually precluded understanding their experiences of pain and distress connected with surgery on the part of the health care workers, who had great difficulties in interpreting the child's gestures and body signs. The parents, however, was always looking for bodily expressions signaling pain, discomfort, frustration and so on, based on their previous knowledge of body language and similar situations. Body language expresses the emotional situation, a dimension of essential importance to learn about concerning children without speech. However, in the first phases of the postoperative period, it was also very difficult for the parents to judge the pain experience and possible fear of the child. Because these parents were especially close to their children, they became perhaps even more sensitive than usual to their children's bodily reactions. They almost felt their supposed pain and distress in their own bodies. In the recovery unit, this embodied connection to their child was coupled with feeling of being utterly helpless.

When a scoliosis—surgery—or a proximal femoral resection is performed, which was the case for children without speech in our study, it means a postoperative period with many parameters to observe: circulation, respiration, bleeding, and pain. In the recovery unit, for instance, when they woke up from anesthesia, these children could neither understand what was going on, nor communicate in words their fear, distress, or pain. They got painkillers (morphine), went to sleep, and woke up again to unfamiliar sounds, to more pain and so on. The parents felt they had placed their child in an unbearable situation, in which they could not do anything to help. A mother said: “We have permitted our child to go through this operation. In a while we will see the result, but now (in the recovery unit and just after leaving the recovery unit) it is just awful!”

Understanding the feelings of distress these parents described seemed to require being aware of the ambivalence they felt about the surgery. For the parents, surgery seemed legitimate, but perhaps not legitimate enough. They took responsibility for letting their child undergo surgery, and wanted responsibility for their child's wellbeing, but needed health professionals not only for support but also to relieve them from some of the responsibility they felt. For instance, the parents of one child who could not speak said: “I have agreed to surgery, and I must take responsibility for the pain my child is undergoing”. They asked themselves questions like: “What is he or she trying to tell me? Do I interpret this or that sign correctly?” and “Did I take the right decision?” One father expressed his worries about pain and medication like this:

Perhaps my child got too little painkiller, because I was afraid to give her too much, as the nurses always asked me: are we going to give her some painkillers? Do you think she has pain now? It is too much for me to take that responsibility. I do not have enough knowledge; I can be wrong.

He wanted to be there for his child. At the same time, being given full responsibility for the child's wellbeing was simply too much. Another father said: “It is difficult for us as parents to understand what's going on because our child cannot speak. For health professionals it is simply impossible to understand.” This utterance might indicate that parents’ strongly felt the need for health care workers whose competence was coupled with an understanding of how complex the situation could be for them. A mother captured the desperation parents of children without speech seemed to feel:

It is tough to be given the responsibility; we have to stay awake all the time. I feel very tired, even more than I thought … Interventions like this are perhaps too much for her, as she cannot speak and has impaired hearing and sight and everything. We simply cannot prepare her for anything at all.

### Sensing bodily reactions

All of the parents described sensing their children's bodily reactions at the hospital as deeply intertwined in the caring for their children, as well as implying frustration and distress. Additionally, they described a need for health care workers who sensed their children's as well as their own (the parents’) bodily reactions.

In the recovery unit, parents felt stressed and very sensitive to their child's bodily reactions, not only the parents of the children without speech. First, they felt anxious, sitting there for hours just waiting while their child was in the operating room. When the child finally entered the unit, parents observed him or her for new hours of worry. When the child entered the recovery unit, a huge operation had been accomplished, lasting for 3–7 h, which affected the child in several ways. Parents explained that CP, with sensory impairment and comorbidity, often including epilepsy, makes a child very sensitive, for instance, to alarms. This was the case for most of the children in our study. A mother said: “Just when my child had gone to sleep, the alarm was ringing intensely, and the child woke up again, confused and scared.”

Children who had stayed at the recovery unit for several hours went back to the hospital ward. Then they had to be mobilized, which meant lots of new stress for the child and the parents. A mother said:

I left the room when my child had to exercise in bed with the physiotherapist. It was simply too great a burden for me to see how painful it was for him. When I came back, he told me he felt betrayed when I left. He was afraid of everything that would happen to him.

The exercise situations after surgery meant pain and stress. Awareness of the child's bodily reactions might positively influence how exercise was performed. Parents strongly expressed that health professionals urgently needed to ally with parents on this issue as well as other important issues, as one father emphasized:

Health professionals have to listen to the parents when they are interpreting the child. For example, when hospitalization arouses feelings of anger and fear, they have to plan the intervention together with the parents and at least prepare both the parents and the child for what is going to happen.

When parents were at the hospital, they felt responsible for making the child's environment as secure as possible and tried to be the ones their children knew from home. When the parents felt emotionally upset and frustrated, however, perpetually afraid something bad might happen to their child, staying calm was not easy. One mother said:

I stayed with her in the recovery unit. I was shocked when I saw her, more shocked than I thought I would be. Her face was swollen, and I hardly recognized her. I was afraid there were complications, but I was assured that everything was normal.

Postoperatively, parents were not always prepared for the consequences of the extensive surgery their child had gone through. One mother said that her child needed treatment in a negative pressure ventilator because he had lost substantial blood and was not breathing adequately, which represented a shock. They had never thought it possible and were glad that they did not know in advance. A mother explained: “We experienced lots of stress. I became depressed and needed long-term sick leave. It reminded me of the newborn period in the neonatal intensive care unit, when I was not prepared.” Parents found it difficult to tell health professionals about their emotional and bodily reactions. Nevertheless, they needed support in the situation, again an urge to be seen and comforted. Parents wanted professionals to be sensitive towards their reactions as well. A father pointed out: “The health care workers are nice to us, but nobody is asking: How do you feel? This is how it is to be parents to a disabled child. Perhaps it had been a good thing for us with a ‘safety valve’?” He is comparing the extremely demanding situation at hospital with the situation of being parents to a disabled child generally, as he sees it.

## Discussion

This study gives insight into the lived experience of some parents of children with CP undergoing surgery. We want to deepen the understanding of core meanings described above, leaning on relevant theory and previous research. Finally, methodological considerations follow.

### Deepening the understanding of core meanings in the essential theme

The parents felt vulnerable in encounters with health professionals at the hospital and discovered how complex the pre- and postoperative processes were. Establishing trust in these vulnerable situations was essential. To establish trust seemed to imply being met at an existential level, which was grounded in an urge for health professionals to show a deeper concern about caring for the child and for the parents. According to Løgstrup (1997), meeting other people with confidence is basic. People contribute to shaping one another's worlds, not by theories and views but by their attitudes toward each other. Meeting each other with confidence means revealing oneself, Løgstrup claims, in the scope and hue of the world (1997, p. 18). Accordingly, a feeling of being rejected follows when the other person turns down one's confidence. Understanding what was at stake for these parents when staying with their child at hospital requires insight in their experience of these situations as extremely demanding and ambivalent. In this light, not feeling listened to and feeling rejected as to knowledge about their child, were experienced as dramatic indeed, which increased feelings of helplessness in a situation they could not escape.

Our findings are in line with findings in a study by Bø and Olsen (2008), where parents to a disabled child emphasized the influence of health professionals being forthcoming and sympathetic, hitting “the right note” in the encounters. At the same time, it was of great value that the health professionals showed real involvement in the child, taking time to be engaged in him/her, and taking an interest in knowing about the history from previous hospitalization and so on. Similar findings were presented in a study about children with cystic fibrosis and their parents. When the parents met a physician who thought he knew everything, without even having met them before, trust was not established. And the opposite, in situations when they met one who was acquainted with their “case”, took interest in their present situation, and cared not only for the child but for the a family as well, they felt taken seriously and comforted (Gjengedal, Rustøen, Wahl, & Hanestad, 2003).

Van Manen (1997) points to four existential levels, one of which is lived space: “… and yet, we know that the space in which we find ourselves affects the way we feel. Home comprises a special space experience, which has something to do with the fundamental sense of our being. Home is where we can be what we are” (1997, p. 102). A person's experiences are different depending on where they are: at home, at work or at hospital. The parents of a child with CP at hospital have to stay there day after day, which strongly affects how they feel and think. They cannot escape the situation. The parents in our study described being exhausted and very vulnerable. In addition, the past influences the present. The parents found themselves in an existential crisis when they saw their child in the postoperative condition, which reminded them of similar situations in the past. They felt a loss of control, scared and lonely when staying at the hospital, which seemed to prevent them from gradually feeling more at home in the situation, even though they stayed there for quite a long time. Instead, doubts about whether surgery was needed and whether the child was cared for properly tormented them, especially in cases where the child could not speak. Hopia et al. (2005) and Haines (2005) emphasized that parents often feel insecure and helpless when their children with disabilities are hospitalized and in need of positive feedback on their parenthood. Hopia et al. (2005) found that families under constant stress needed special support, and underscored that high-risk families must be identified and offered professional help. The parents in our study had comprehensive demands regarding caring for their disabled child on a daily basis. Surgery meant an increased burden, lasting for weeks and months. In a study about adolescents with CP undergoing spinal surgery, Salisbury, LaMontagne, Hepworth, and Cohen (2007) found that a predominant stressor was parents’ feelings of loss of their parental role after surgery. Parents were distressed because they felt helpless, especially about the information given to the children. In our study, parents clearly expressed that determining the children's wellbeing was highly problematic, as they could not express their feelings and discomforts. Bø and Olsen (2008) showed that the parents of children with CP had to fight for the interests of their children, and encountered negative attitudes occasionally in various situations, as in the hospital.

In lived time and space, parents were aware of their history as parents of a child who could not speak. It is complicated and not fully possible to understand children without speech. These children also had muscle spasms, which made it hard to read their body language. However, it is not unusual that parents of a child with CP can interpret their child's bodily reactions and signs to quite a great extent, a competence that has evolved over the years. As such, they are normally able to sense if the child is in distress. Nevertheless, in this unusual situation, especially in the intensive care unit and just after leaving this unit, they did not know what reactions could be considered “normal” and not. They had been at the hospital before, and were deeply aware that their children differed from healthy children and from disabled children without mental retardation undergoing surgery. The hours and days bedside felt long, very long. Lived time appears to slow down when people feel anxious, in despair or in pain (van Manen, 1997). At the hospital, many factors increased the feeling of time slowing down. Parents had to stay there observing and being aware of the child. The parents had more difficulty than usual in understanding what the child communicated in his or her unfamiliar health condition after surgery and in the unfamiliar world at hospital. The situation stirred up stronger than expected feelings of helplessness and doubt about the legitimacy of the surgery. Nevertheless, who could be more experienced at interpreting the child's condition than the parents?

The strongly felt need for awareness of both the child's and the parent’ bodily reactions arose in this context. Findings from previous research (Maciver, Jones, & Nicol, 2010) indicate that parents of children with complex regional pain syndrome might require support to master fearful emotional reactions when they are involved in managing their children's pain. The parents of children who could not speak in our study were in an even more demanding situation, simply because their children were mentally retarded and without speech. They felt huge responsibility and were in a dilemma, because they were not professionals and yet were responsible for determining when to give painkillers. Thus, prior experience was not sufficient to guide them in the new situation. These experiences imply that health professionals need to emphasize developing a parent–provider relationship that informs parents of normal reactions and needs after undergoing surgery, and simultaneously acknowledge the competence of parents as experts knowing their children. Bø and Olsen (2008) described what they termed as “the power of parents”. It means a power growing out of the parents’ competence on and sensitivity towards their disabled child, just because they are the people who know their child best of all. This “expert” sensitivity towards the child is also what contributes substantially to the parents’ vulnerability.

People live in different contexts, and embody different histories. Understanding how a person thinks and feels requires knowing the person's history and their significant concerns, according to Benner and Wrubel (1989), anchoring their thoughts in the phenomenological tradition. Accordingly, listening to the parents’ stories makes a difference as to understanding parents’ reactions. Sensitivity is needed in order to be aware of and interpret the expressions of the whole person. In this study, when the parents saw their child in the recovery unit, they expressed memories and reactions of situations from the past, when the child had been at the hospital and they had been afraid of losing her or him. From the viewpoint of the lived body, van Manen (1997) claims that parents experience their children as separate but also physically very close. This means that, when a child undergoes surgery, there may be “a deep significance in the knowledge that parents and children are of one flesh” (p. 105). When the child is ill and needs his or her parents in a special way, the parents’ experiences and reactions should be understood from this perspective.

### Methodological considerations

Hermeneutic phenomenological studies establish a close relationship between the researcher and the respondents, enabling participants to share lived experience and subjective meanings. In such conversations, the researcher can mobilize participants to reflect on their experiences retrospectively, to explore their deeper meanings (van Manen, 1997). Qualitative methodology, such as in-depth interviews in a phenomenological research design, is demanding. They require sensitive interpretive skills, creative talents and they constantly seek an open and dwelling attitude (Dahlberg, Nystrøm, & Drew, 2008; Kvale, 2005; van Manen, 2006). During these interviews, the first author was inspired by and able to get insight into the parents’ lived experience at the hospital. The participants invested more than a passing interest in the research project by willingly involving themselves.

However, to get at deeper meanings of a specific phenomenon, researchers have to give a sense of direction by asking questions, which content and meaning always contribute in shaping the answers (Dahlberg et al., 2008). The interviewer's insight, skills, involvement and critical reflection are important. By asking and searching with openness and thoroughness, researchers may get unique insights into, for instance, being the parents of a child with CP undergoing surgery. Reflexivity also involves considering the importance of one's own viewpoint in the process. People who are too close to the field may have problems in critical reflection underway. The first author became emotionally involved in the parents’ stories, and was challenged to use and question her pre-understandings in the field. By listening to the tapes, reading and re-reading the interview texts, discussing with the co-researchers, as well as writing and re-writing, the first author was able to move between closeness and distance, to practice wonder and thoughtfulness, and constantly working on being open to discover a deeper understanding of the texts. According to pre-understanding, researchers have a lifeworld that comprises the inescapable context for the research (Dahlberg et al., 2008).

According to Stige, Malterud, and Midtgarden (2009), reflexivity is not a matter of methodological control but has a dialogical structure of questions and answers throughout the research process, because each researcher brings unique skills and commitment to her or his research topic. Several participants stated that the interview was the first time they had the opportunity to talk freely about the experiences and feelings they had at the hospital, which implies that they felt listened to in the interview situation.

### Implications for nursing practice

Our study fills a gap in the knowledge about the lived experience of parents with a child with CP undergoing surgery. The findings indicate the importance of acknowledging parents’ situation as extremely vulnerable and demanding, and the importance of nurses to create trust in the relation and ally with the parents. We need to approve and take advantage of the parents’ competence as experts in interpreting their children's needs and reactions in acute events or situations involving specialist care services, such as surgery. Nurses at the unit being sufficiently aware of the unique competence of the parents of children with CP may contribute to higher-quality collaboration between the parties, higher quality of care and less suffering for the child and family. Nevertheless, parents should not be given too much responsibility for evaluating the child's health. Nurses should balance giving and freeing parents from responsibility. Their managers should equip their specialist intensive care nurses with more knowledge about what is at stake from the parents’ point of view in critical situations for their child undergoing surgery, as well as defining and developing routines for improving communication skills.

## References

[CIT0001] Åkerstedt A, Risto O, Ødman P, Øberg B (2010). Evaluation of single event multilevel surgery and rehabilitation in children and youth with cerebral palsy – a 2 year follow up study. Disability and Rehabilitation.

[CIT0002] Benner P, Wrubel J (1989). The primacy of caring.

[CIT0003] Bø B, Olsen R. B (2008). Utfordrende foreldreskap [Challenging parenthood].

[CIT0004] Brinchmann B. S, Førde R, Nortvedt P (2002). What matters to the parents?. Nursing Ethics.

[CIT0005] Dahlberg K, Drew N, Nystrøm M (2008). Reflective lifeworld research.

[CIT0006] Fog J (2004). Med samtalen som utgangspunkt: det kvalitative forskningsinterview.

[CIT0007] Gjengedal E, Rustøen T, Wahl A, Hanestad B (2003). Growing up and living with Cystic Fibrosis. Advances in Nursing Science.

[CIT0008] Haines C (2005). Parents’ experiences of living through their child's suffering from and surviving severe meningococcal disease. British Association of Critical Care Nurses, Nursing in Critical Care.

[CIT0009] Hall E. O. C (2005). Being in an alien world: Danish parents’ lived experiences when a newborn or small child is critically ill. Scandinavian Journal of Caring Sciences.

[CIT0010] Holaday B, Hallstrøm I, Runesson I, Elander G (2002). Observed parental needs during their child's hospitalization. International Pediatric Nursing.

[CIT0011] Hopia H, Paavilainen E, Åstedt-Kurki P (2005). The diversity of family health: Constituent systems and resources. Scandinavian Journal of Caring Sciences.

[CIT0012] Hopia H, Tomlinson P (2005). Child in hospital: Family experiences and expectations of how nurses can promote family health. Journal of Clinical Nursing.

[CIT0013] Huang Y.-P, Kellett U. M, St. John W (2010). Cerebral palsy: Experiences of mothers after learning their child's diagnosis. Journal of Advanced Nursing.

[CIT0014] Iversen A, Graue M, Clare J (2009). Parents’ perspectives of surgery for a child who has cerebral palsy. Journal of Pediatric Health Care.

[CIT0015] Jones J, Borbasi S, Clare J, Hamilton H (2003). Interpretive research: Weaving a phenomenological text. Writing research: Transforming data into text.

[CIT0016] Knaus A, Terjesen T (2009). Proximal femoral resection arthroplasty for patients with cerebral palsy and dislocated hips. Acta Ortopedaedica.

[CIT0017] Kristensson-Hallström I (2000). Parental participation in pediatric surgical care. AORN Journal.

[CIT0018] Kvale S (2005). Producing knowledge through interviews.

[CIT0019] Løgstrup K (1997). The ethical demand.

[CIT0020] Maciver D, Jones D, Nicol M (2010). Parents’ experiences of caring for a child with chronic pain. Qualitative Health Research.

[CIT0021] McCullough N, Parkes J, Kerr C, Mc Dowell B (2011). The health of children and young people with cerebral palsy; a longitudinal, population-based study. International Journal of Nursing Studies.

[CIT0022] McKearnan K. A, Kieckhefer G. M, Engel J. M, Jensen M. P, Labyak S (2004). Pain in children with cerebral palsy: A review. Journal of Neuroscience Nursing.

[CIT0023] Merleau-Ponty M (1996). Phenomenology of perception.

[CIT0024] Murphy N, Hoff A, Jorgensen C, Norlin T, Firth C, Young C. P (2006). A national perspective of surgery in children with cerebral palsy. Pediatric Rehabilitation.

[CIT0025] Nystrøm M, Dahlberg K (2001). Pre-understanding and openness – a relationship without hope?. Scandinavian Journal of Caring Sciences.

[CIT0026] Peden-McAlpine C, Tomlinson P, Forneris S, Genck G, Meiers S (2005). Evaluation of a reflective practice intervention to enhance family care. Journal of Advanced Nursing.

[CIT0027] Salisbury M, LaMontagne L, Hepworth J, Cohen F (2007). Parents’ self-identified stressors and coping strategies during adolescents’ spinal surgery experiences. Clinical Nursing Research.

[CIT0028] Secco L, Askin D, Ecco L, Yu C. T (2006). Factors affecting parenting stress among biologically vulnerable toddlers. Issues in Comprehensive Pediatric Nursing.

[CIT0029] Stige B, Malterud K, Midtgarden T (2009). Toward an agenda for evaluation of qualitative research. Qualitative Health Research.

[CIT0030] van Manen M (1997). Researching lived experience: Human science for an action sensitive pedagogy.

[CIT0031] van Manen M (2006). Writing qualitatively, or the demands of writing. Qualitative Health Research.

[CIT0032] Vetlesen A. J, Stanicke S (1999). Fra hermeneutikk til psykoanalyse [From hermeneutics to psychoanalysis].

[CIT0033] Wigert H, Berg M, Hellstrøm A (2007). Health care professionals’ experiences of parental presence and participation in neonatal intensive care unit. International Journal of Qualitative Studies on Health and Well-being.

